# Sacubitril-Valsartan Compared With Enalapril for the Treatment of Heart Failure: A Decision-Analytic Markov Model Simulation in China

**DOI:** 10.3389/fphar.2020.01101

**Published:** 2020-07-23

**Authors:** Yue Wu, Shuo Tian, Peipei Rong, Fan Zhang, Ying Chen, Xianxi Guo, Benhong Zhou

**Affiliations:** ^1^Department of Pharmacy, Renmin Hospital, Wuhan University, Wuhan, China; ^2^School of Pharmaceutical Sciences, Wuhan University, Wuhan, China

**Keywords:** sacubitril-valsartan, enalapril, cost effectiveness, heart failure, China

## Abstract

**Objectives:**

Heart failure with reduced ejection fraction (HFrEF) is a major health concern globally due to high mortality rates, frequent hospitalization and considerable medical expenditure. The prevalence of HFrEF is steadily rising in Asian countries, and populous, developing countries like China are facing a significant socio-economic burden as a result. Sacubitril-valsartan (Sac-Val) is currently a class I recommendation for treating HFrEF in major guidelines, although it has not been pharmaco-economically evaluated in China. To this end, we compared the cost-effectiveness of Sac-Val and enalapril based on the negotiated prices in order to fully assess the expected costs and benefits of the clinical use of Sac-Val in China.

**Method:**

A Markov model was constructed to estimate long-term clinical and economic outcomes of Sac-Val versus enalapril for HFrEF patients in China over a 10-year horizon. Primary model outcomes were total costs and quality-adjusted life years (QALYs), and the incremental cost-effectiveness ratio (ICER).

**Results:**

Treatment with Sac-Val resulted in 4.67 QALYs at the cost of $4,684.25, while enalapril yielded 4.40 QALYs at the cost of $4,014.47. Compared to enalapril, Sac-Val was associated with a gain of 0.27 QALYs, resulting in an ICER of $ 2,480.67 per QALY. Deterministic sensitivity analysis showed robust results. Probabilistic sensitivity analysis suggested that Sac-Val has a 99.99% probability of being cost-effective at the willingness-to-pay threshold of $10,276.

**Conclusion:**

From Chinese patients’ perspective, Sac-Val is a cost-effective treatment option for HFrEF in China compared to enalapril. Our findings can aid clinicians plan the Sac-Val regimen, as well as decision makers to discuss the value and position of novel angiotensin receptor neprilysin inhibitors (ARNIs) in future.

## Introduction

Heart failure (HF) is a major health concern worldwide due to the elevated mortality rates, frequent hospitalization and high expenditures (Savarese and Lund, 2017). Globally, HF affects an estimated 38 million people, with a 1%–2% prevalence in the adult population (Braunwald, 2015). Depending on extent of the decline in left ventricular ejection function (LVEF), HF is typically classified into heart failure with preserved ejection fraction (HFpEF) and with reduced ejection fraction (HFrEF) types. However, patients with HF have poor prognosis regardless of the LVEF, with a poor survival rate of < 50% within 5 years after the first hospitalization (Tribouilloy et al., 2008; Buddeke et al., 2020). In addition, almost half of the HF patients suffer from fatigue, breathlessness, edema or arrhythmias, which significantly reduces the quality of life (Gohler et al., 2009). HF is also associated with high rates of hospitalization and readmission (Cook et al., 2014), and accounts for 1%–2% of the overall hospitalizations in USA and Europe (Ambrosy et al., 2014).

The etiology of HF is variable depending on the geographical region but hypertension and coronary artery disease are the major risk factors. Regular physical activity and maintaining a healthy BMI can lower the risk of HF or at least slow disease progression (Horwich and Fonarow, 2017). However, majority of the HF patients require drug therapy, especially those with significant loss of cardiac function. ACE inhibitors (ACEIs) are typically the first-line treatment for HFrEF over the past few decades. Despite that ACEIs reduce the risk of hospitalization and death, the rates of hospitalization and death remain high as well as the loss of quality of life in HF patients (McMurray et al., 2018). The combination of the neprilysin inhibitor sacubitril and angiotensin-II receptor blocker (ARB) valsartan (Sac-Val) was proposed as a promising alternative for treating HFrEF in the last decade. PARADIGM-HF, a multinational phase III, double-blind, prospective randomized clinical trial (McMurray et al., 2014), showed that Sac-Val was superior to enalapril in terms of reducing the composite primary outcome of cardiovascular (CV) death or hospitalization in HFrEF patients. Based on their findings, Sac-Val is now a class I recommendation in the recent guidelines for the management of HF in USA (Yancy et al., 2017), Europe (Ponikowski et al., 2016) and China (Chinese Medical Association et al., 2019) alongside other standard therapies.

Apart from the therapeutic effects, the results from PARADIGM-HF have also been used for evaluating the economic viability of Sac-Val (Liu et al., 2020), especially in the developed countries (Gaziano et al., 2016; King et al., 2016; Sandhu et al., 2016; van der Pol et al., 2017; Zanfina et al., 2017; Gandjour and Ostwald, 2018; Krittayaphong and Permsuwan, 2018; Liang et al., 2018; Zueger et al., 2018; Park et al., 2019; van der Pol et al., 2019). Owing to the high willingness-to-pay (WTP) threshold, Sac-Val is likely to be cost-effective compared to enalapril in United States (Gaziano et al., 2016; King et al., 2016; Sandhu et al., 2016), United Kingdom (McMurray et al., 2018) and some other European countries (Zanfina et al., 2017; Gandjour and Ostwald, 2018; McMurray et al., 2018; van der Pol et al., 2019) despite the higher acquisition cost. However, few studies have evaluated the pharmaco-economics of Sac-Val in the low and middle-income Asian countries.

China is the largest developing country in Asia with a population of 1.3 billion, and is now facing a heavier socio-economic burden of HF compared to the West. With a steadily aging population, and increasing incidence of cardiovascular risk factors like obesity, hypertension, and diabetes, the prevalence of HF has significantly risen in China in recent years (Huang et al., 2017; Zhang et al., 2017). Due to the large population and limited economic resources, China has more HF patients with lower capacity for bearing the costs of medical treatment. Furthermore, the clinical phenotypes and treatment patterns also vary considerably between the developed and developing countries, which usually translates to similarly poor or even worse outcomes in Asian population (Mentz et al., 2016). Given that pharmaco- economic evaluation is greatly affected by the economic capacity and patient subgroups, the expected costs and benefits of Sac-Val treatment in China remain unknown.

In 2017, in order to further improved the quality of medical care, the Chinese government launched the national negotiation on the prices of groups of drugs covered by medical insurance. Sac-Val was listed in the negotiating drug catalog in 2019, and is covered by the national insurance reimbursement, which could greatly increase its affordability. In this study, we compared the cost-effectiveness of Sac-Val and enalapril based on the current price control mechanism in the Asian subgroup, in order to fully assess the expected costs and benefits of using Sac-Val in China.

## Methods

### Model Structure

**Figure 1:**
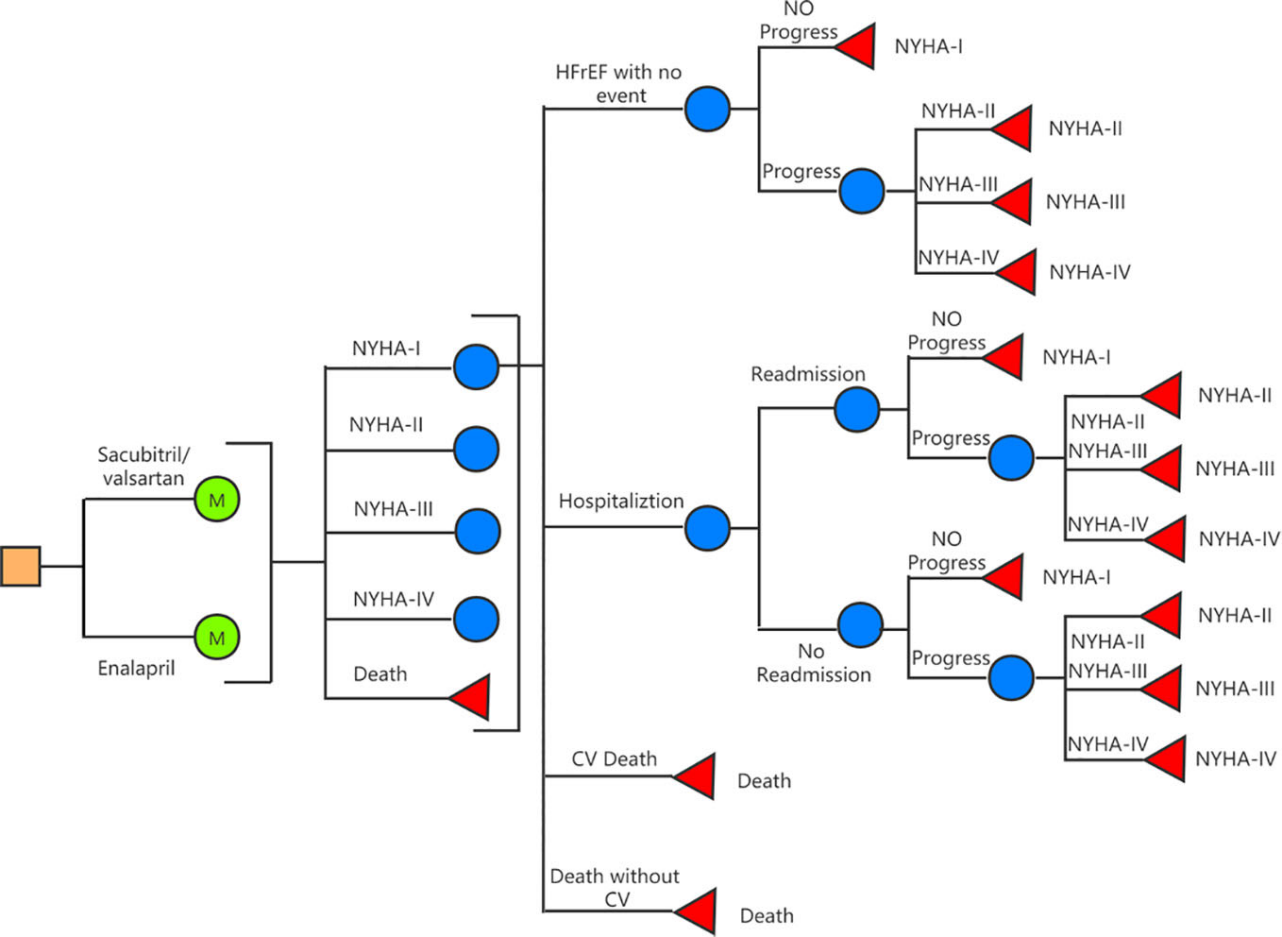
Schematic representation of the Markov model.

A decision-analysis state-transition Markov model with one-month cycles and 10-year horizon was created to compare Sac-Val versus enalapril for HFrEF patients in China (**Figure 1**). Five Markov states—including the New York Heart Association (NYHA) classes I to IV and deaths—were defined in the model. Within-cycle transitions state included HF hospitalization, 30-day readmission, progression to subsequent NYHA class, cardiovascular (CV) death and non-CV death. The assumption was made that during each cycle, patients may either remain in their current health state or transition to the next state due to an event. The starting age of the patient cohort was set at 64 years (Liu et al., 2014) in accordance with the mean age of Chinese population with HF. The probability of beginning the first cycle in a given NYHA class was determined according to the characteristic of PARADIGM-HF at the time of randomization (4.5% NYHA I, 71.6% NYHA II, 23.1% NYHA III, 0.8% NYHA IV) (McMurray et al., 2014). A 10-year time horizon was used based on average life expectancy of 5 to 10 years for HF patients (Allen et al., 2008; Alter et al., 2012). Model construction and analyses were performed using TreeAge Pro SuiteTM software 2019 (Williamstown, MA. USA).

### Date and Sources

#### Probability

A targeted literature review was conducted to identify appropriate model inputs (**Table 1**), and the model was developed in a hypothetical cohort based on the characteristics observed in PARADIGM-HF trial. The baseline probabilities of hospitalization and CV death were derived from the Kaplan-Meier curves of enalapril group. Weibull and Exponential distribution were fitted to obtain the individual monthly transition probabilities (Liang et al., 2018). The clinical benefits of Sac-Val were modeled by applying the hazard ratios (HRs) to the baseline probabilities, and the NYHA class-specific probabilities for hospitalization and CV deaths were obtained by applying the hazard ratios (HRs) and relative risks (RRs) to the baseline probabilities. Age-dependent non-CV mortality was derived from local epidemiological data (2018) after excluding CV deaths (**Table 2**). The readmission rate of 16.23% was derived from the national insurance database including 7,847 patients with HF (Huang et al., 2017). Readmission rates were assumed to be the same in both arms because no significant difference was observed in Asia-Pacific subgroup of PARADIGM-HF (Desai et al., 2016). NYHA progressing probabilities in Sac-Val and enalapril treatment arms were 0.0068 and 0.0088 as reported previously (Zueger et al., 2018). NYHA class specific transition probabilities were cited from an established matrix (King et al., 2016) (**Table 3**), and were assumed to be fixed in one-month cycle and same in both treatment arms, since the effect of Sac-Val on NYHA class transition relative to that of enalapril is unclear.

**Table 1:** Parameters inputs.

Input variable	Vaule	Low	Up	Distribution	Source	Notes
**Baseline Probability**						
Weibull model for hospitalization in enalapril	λ=−0.00097; γ= 1.02685	0.00868	0.0145	–	(McMurray et al., 2014; King et al., 2016)	Extrapolated from published Kaplan-Meier curves from PARADIGM-HF trial
Exponential model for CV death in enalapril	λ=−0.00577	0.00412	0.00844	–	(McMurray et al., 2014; King et al., 2016)	
Readmission	0.0147	0.0132	0.0161	Beta	(Desai et al., 2016)	± 10% of the mean
NYHA Progress for enalapril	0.0088	0.0079	0.0097	Beta	(Zueger et al., 2018)	± 10% of the mean
NYHA Progress for Sac-val	0.0068	0.00612	0.00748	Beta	(Zueger et al., 2018)	± 10% of the mean
Background no-CV mortality	Aged specific				(National Health Commission of the People’s Republic of China, 2018)	–
Hazard ratios (HR)/Risk ratio(RR) compared to baseline*						
HR of Hospitalization for Sac-val	0.79	0.71	0.89	Log-normal	(McMurray et al., 2014)	95% CI
HR of CV death for Sac-val	0.80	0.71	0.89	Log-normal	(McMurray et al., 2014)	95% CI
HR of Hospitalization for NYHA III	1.71	1.33	2.18	Log-normal	(Ahmed et al., 2006)	95% CI
HR of Hospitalization for NYHA IV	3.4	1.69	6.84	Log-normal	(Ahmed et al., 2006)	95% CI
RR of CV death for NYHA III to baseline	1.372	1.303	1.445	Log-normal	(Pocock et al., 2012)	95% CI
RR of CV death for NYHA IV	1.640	1.503	1.790	Log-normal	(Pocock et al., 2012)	95% CI
**Utility inputs**						
NYHA I and II	0.780	0.741	0.819	Beta	(Xuan et al., 2017)	± 5% of the mean;
NYHA III	0.715	0.679	0.751	Beta	(Xuan et al., 2017)	± 5% of the mean;
NYHA IV	0.660	0.627	0.693	Beta	(Xuan et al., 2017)	± 5% of the mean;
Disutility (one-time)						
Hospitalization	−0.1	−0.13	−0.08	Beta	(King et al., 2016)	95% CI
Readmission	−0.1	−0.13	−0.08	Beta	(King et al., 2016)	95% CI
**Cost inputs**						
Sac-Val	17.12	13.70	30.46	Gamma	Local date	Local date
Enalapril	10.65	0.90	12.78	Gamma	Local date	Local date
Outpatient visit	41.56	33.26	49.87	Gamma	Local date	± 20% of the mean;
Copay ratio for inpatient	0.3	0.1	1	–	Local date	–
Cost of events (one-time)						
Hospitalization	1,920.49	1,536.39	2,304.59	Gamma	(Huang et al., 2017)	± 20% of the mean;
Readmission	1,340.05	1,072.04	1,608.06	Gamma	(Huang et al., 2017)	± 20% of the mean;

*The baseline probabilities were assumed to the probabilities of NYHA class I and II.

**Table 2:** Background no-cardiovascular (CV) mortality.

Age	Background no-CV mortality	Age	Background no-CV mortality
1	0.002949	50	0.0011998
5	0.000284	55	0.0029394
10	0.00015	60	0.0028332
15	0.000169	65	0.0066972
20	0.000177	70	0.0097194
25	0.000154	75	0.0121676
30	0.000298	80	0.016739
35	0.000458	85	0.0331037
40	0.000472	100	1.00
45	0.00071		

**Table 3:** New York Heart Association (NYHA) progressing probabilities per one-month cycle.

From NYHA	To HYHA			
	Class I	Class II	ClassIII	Class IV
Class I	–	0.831	0.169	0
Class II	0.422	–	0.531	0.047
Class III	0	0.847	–	0.153
Class IV	0	0	1.000	–

#### Cost and Utilities

Health utility values described the quality of life for each health states were obtained from published studies (**Table 1**) and were scored according to a scale ranging from 0 (death) to 1 (perfect health). One-time dis-utilities of −0.1 was used for each hospitalization and readmission event in NYHA I-IV (King et al., 2016). Consistent with the perspective of the Chinese patients, the model only incorporated the direct healthcare costs of HF therapy, and a half-cycle correction was applied in the model. Drug costs were derived from the prices charged for 10mg enalapril twice daily and 200 mg Sac-Val twice daily (**Table 4**) in China. Considering the differences in drug quality between foreign and local manufacturers, the generic price of enalapril produced by overseas pharmaceutical companies was used in base case analysis. The costs for HF-associated hospitalization, readmission and doctor visits were derived from local epidemiological data (Huang et al., 2017), and assumed to be equivalent across both treatment groups. All the costs inputs were inflated to 2019. Since that the discount rate is not explicitly recommended in China, the usual discount rate of 3.5% per year was used to eliminate the effects of inflation.

**Table 4:** Drug dose and costs.

Drug	Dose	Unit Price ($)	Medicare copay ratio^*^	Monthly price($)	Monthly cost($)
Sac-val	200 mg twice daily	1.42/200 mg	0.8	85.61	17.12
Enalapril	10 mg twice daily	0.18/10 mg	0	10.65	10.65

*Medicare copay ratio for outpatient.

#### Outcomes

The main outcome measured in this analysis was incremental costs per quality-adjusted life-year (QALY) gained, expressed as the incremental cost-effectiveness ratio (ICER). Since there is no fixed WTP threshold currently to determine cost-effectiveness in China, the gross domestic product (GDP) per capita ($10,276) and threefold GDP per capital ($30,828) were used as the threshold. Cost effectiveness was assessed annually to determine at which point in time the treatment options achieved acceptable levels.

### Sensitivity Analysis

One-way, two-way and probabilistic sensitivity analysis (PSA) were performed to assess the impact of parameter uncertainty on the results. Model parameters were varied over their 95% confidence intervals. Variations of ±5%, ± 10%, and ±20% were assumed for parameters of utility, probability and medical costs that have no specified data range. The range of the cost for Sac-Val and enalapril were set based on the distribution of local prices (including all products from foreign and local manufacturers). In PSA, probabilities of clinical outcomes and health utilities were assigned as beta distributions. Log-normal distributions were applied for HRs and RRs. Gamma distributions were assumed for drug and healthcare costs. All variables were allowed to simultaneously vary stochastically in PSA. A second-order Monte Carlo simulation was performed (n=10,000) based on the variable-specific distributions. Results of the PSA were presented graphically as scatterplots and cost-effectiveness acceptability curves.

## Results

### Base-Case Analysis

Over a 10-year horizon, 51.3% patients were predicted to die in the Sac-Val cohort compared with 58.1% in the enalapril cohort. The predicted costs, benefits and ICERs of the two arms are summarized in **Table 5**. Furthermore, treatment with Sac-Val was predicted to yield 4.67 QALYs at the cost of $4,684.25, while enalapril yielded 4.40 QALYs at the cost of $4,014.47. This resulted in an ICER of $2,480.67 per QALY gained for Sac-Val versus enalapril, which is much lower than the cost-effectiveness threshold.

**Table 5:** Base case analysis and one-way sensitivity analysis on time horizon and initial New York Heart Association (NYHA) class distribution.

	Treatment strategies	Cost($)	Incremental cost ($)	QALY	Incremental QALYs	ICER($/QALYs)
Base case	Sac-val	4,684.25	669.78	4.67	0.27	2,480.67
	Enalapril	4,014.47		4.40		
Time horizon						
5-years	Sac-val	2,973.81	362.99	2.95	0.10	3,684.57
	Enalapril	2,610.82		2.85		
15-years	Sac-val	5,661.36	900.47	5.64	0.44	2,032.52
	Enalapril	4,760.89		5.20		
20-years	Sac-val	6,170.73	1,049.23	6.14	0.56	1,874.00
	Enalapril	5,121.5		5.58		
Initial NYHA class distribution:5% I; 20% II; 45% III; 30% IV						
5-years	Sac-val	2,995.24	379.67	2.73	0.09	4,175.26
	Enalapril	2,615.57		2.64		
10-years	Sac-val	4,609.59	687.66	4.33	0.25	2,697.82
	Enalapril	3,921.93		4.08		
20-years	Sac-val	5,977.42	1,044.35	5.69	0.51	2,030.94
	Enalapril	4,933.07		5.17		

Varying the time horizon significantly affected the cost-effectiveness estimates (**Table 5**). At 5 years of follow-up, the ICER per QALY gained was $3,684.57, which decreased to $2,032.52 and $1,874.00 when the time horizon extended to 15 and 20 years respectively. The effect of the initial NYHA class distribution was also calculated, and showed that a higher proportion of NYHA class III and IV patients, in accordance with epidemiological characteristics of HF in China, increased the estimated ICER to $2,697.82 per QALY gained.

### Sensitivity Analysis

#### One-Way Sensitive Analysis

**Figure 2:**
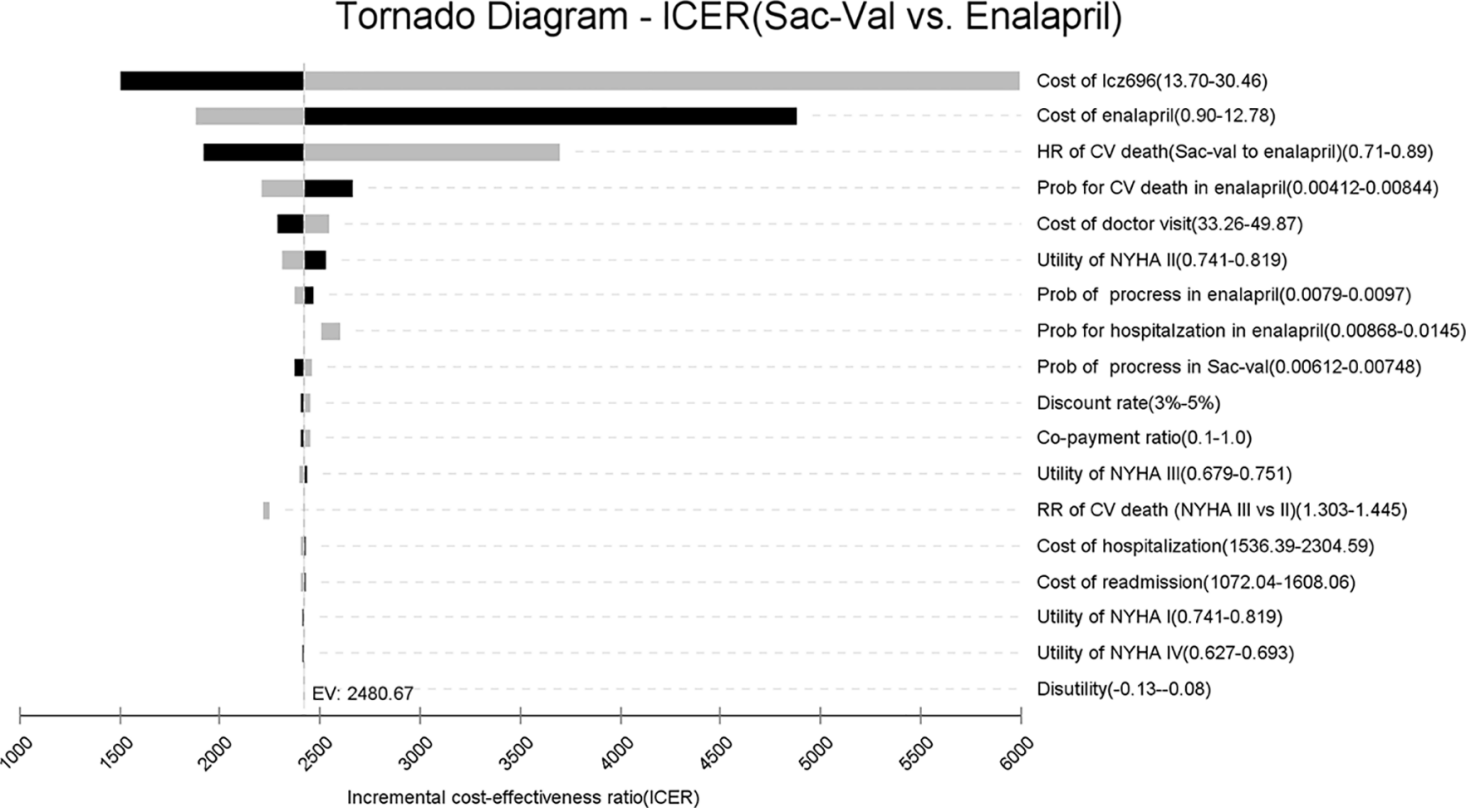
Tornado analysis: ICER of Sac-val vs. enalapril over plausible ranges of model inputs.

A tornado analysis was performed to evaluate the impact of input parameters (**Figure 2**). The prices of Sac-Val and enalapril had the largest impact on ICER, followed by the hazard risk of CV deaths, CV mortality and the cost of outpatient visit. At the highest end of Sac-Val cost or the lowest end of enalapril cost, ICER respectively increased to $5,992.46 or $4,878.37 per QALY. Other input parameters, such as health utilities, therapeutic costs and risks for clinical events, had a minor impact on estimated ICER. Furthermore, the ICER values did not increase over the cost-effectiveness threshold when varying any model inputs across their plausible ranges, indicating that Sac-Val is robustly cost-effective compared to enalapril.

#### Two-Way Sensitive Analysis

**Figure 3:**
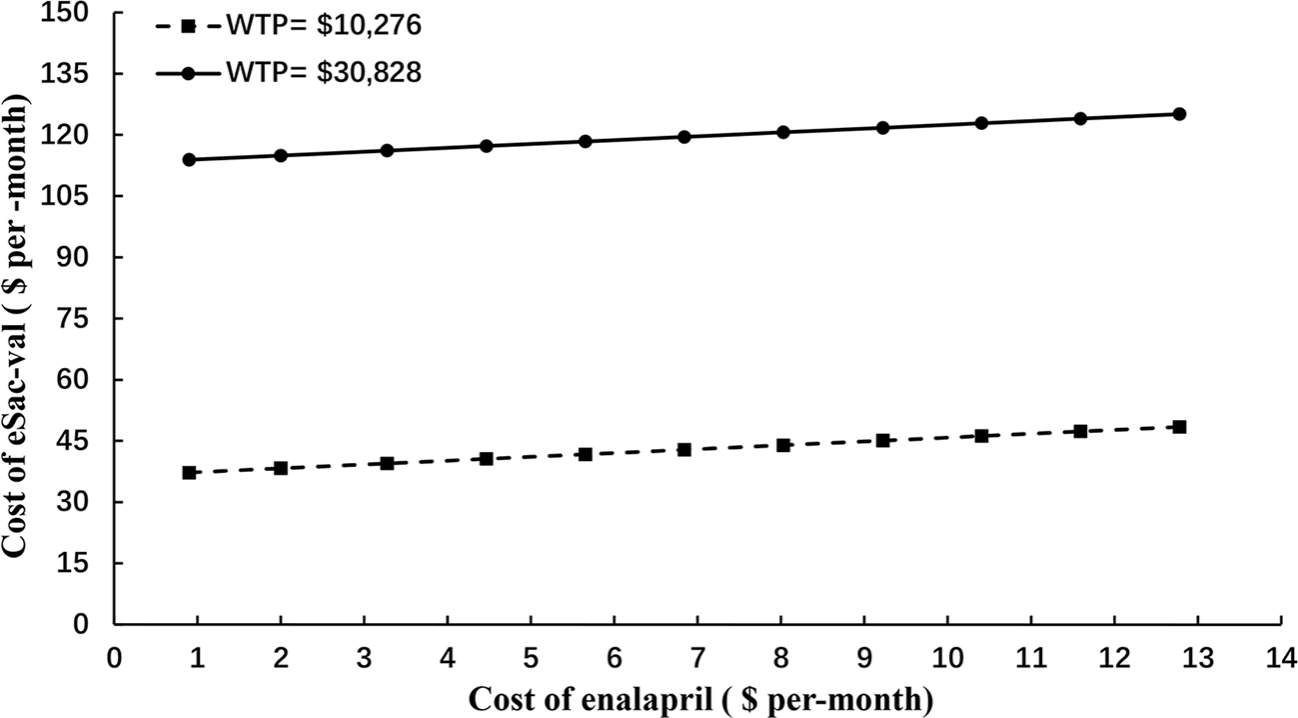
Two-way sensitive analysis on the cost of Sac-val and enalapril.

To fully assessed the potential impact of price negotiation on the outcomes, two-way sensitive and threshold analysis was conducted by extending the cost range of Sac-Val to $150 per-cycle, which is ~500% of the highest end of the cost (**Figure 3**). Based on the current cost of enalapril at $0.9 to $12.78 per-month, Sac-Val will have to cost $37.25–$48.44 per-month to be considered cost-effective with the WTP threshold of $10,276. When threefold GDP per capital ($30,828) was applied as the WTP threshold as recommended by World Health Organization (WHO), the monthly cost of Sac-val could increase to $113.91–$125.10.

#### Probabilistic Sensitivity Analysis

**Figure 4:**
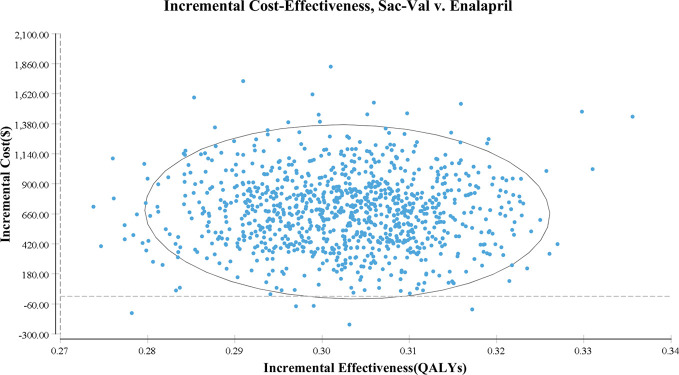
Scatter plot of the probabilistic sensitivity analyses.

**Figure 5:**
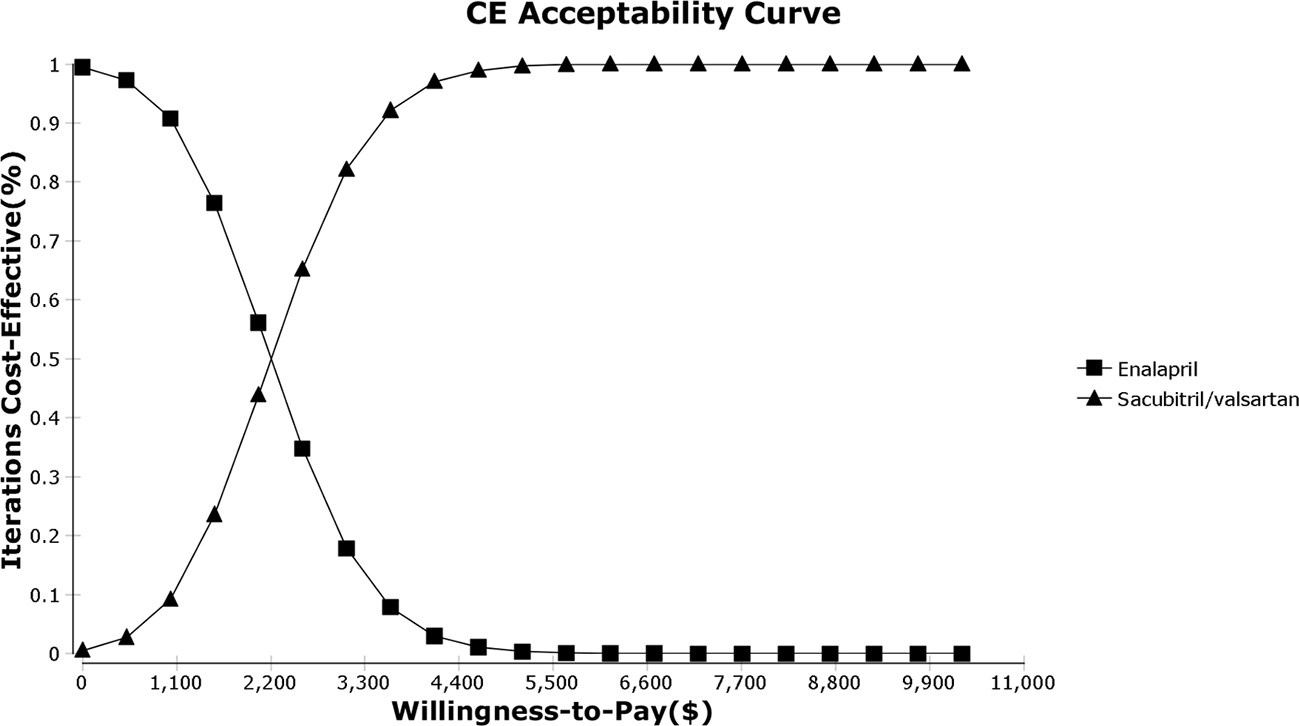
Willingness-to-pay curve.

As shown in the PSA simulation scatterplot in **Figure 4**, Sac-Val had a higher average total cost of $ 4,522.11 (95% CI, $ 3,341.23–$ 5,905.97) compared to $ 3,846.04 (95% CI, $ 2,808.77–$ 5083.80) of enalapril. Over a 10-year horizon, Sac-Val gained average QALYs of 4.21 (95% CI, 4.18–4.23) compared to 4.51 (95% CI, 4.49–4.54) gained by enalapril. A cost-effectiveness acceptability curve was plotted to demonstrate the proportion of simulations that were cost-effective at the WTP values (**Figure 5**). Compared to enalapril, there was a 99.99% probability that Sac-Val is cost-effective using a WTP threshold of $10,276. The cost-effectiveness probabilities did not change significantly when the monthly costs of Sac-Val varied between 80% and 120%. If the costs further increased to $113.91 and $125.10, the probability of cost-effectiveness ranged from 86.2% to 69.7% at a WTP of $30,828/QALY gained.

## Discussion

The prevalence of HFrEF is steadily increasing in Asian countries, resulting in considerable socioeconomic burden due to huge population and limited economic resources. The novel combination drug Sac-Val has yielded clinical benefits in HFrEF patients. Given the low-cost generic status of ACEIs, Sac-Val should be pharmaco-economically evaluated in Asia to determine whether its clinical benefits are worth the additional costs. This is the first study to compare the cost-effectiveness of Sac-Val and enalapril in China based on the health costs and resources, which can further enable informed healthcare decision-making in developing countries.

The Chinese government launched national price negotiation between the National Health Insurance Administration and drug producers in 2017 in order to further improve the quality of medical care. Novel drugs with significant clinical efficacy but high costs can only be included in the medical insurance reimbursement list after the price is reduced. The reimbursement list was renewed on Jan 1, 2020, and included several drugs that can improve patient quality of life, such as Sac-Val. We used the latest negotiated price of Sac-Val to assess its expected costs and benefits in China. Base case analysis showed that Sac-Val is more costly and also more effective compared to enalapril, which is consistent with other cost-effectiveness analyses. In addition, the estimated ICER was significantly lower than the GDP per capital of China regardless of the time horizon, owing to the low negotiated price and insurance coverage. Deterministic sensitivity analysis showed that the results were robust, and probabilistic sensitivity analysis further suggested a 99.99% probability of Sac-Val being cost-effective

Several studies have compared the cost-effectiveness of Sac-Val and enalapril in developed countries. Owing to the high WTP threshold, Sac-Val is cost-effective in countries like United States (Gaziano et al., 2016; King et al., 2016; Sandhu et al., 2016), Netherlands (van der Pol et al., 2017), United Kingdom (McMurray et al., 2018) and other European countries (Zanfina et al., 2017; Gandjour and Ostwald, 2018; McMurray et al., 2018; van der Pol et al., 2019), even with high estimated ICERs. In contrast, opposite conclusions were drawn in similar studies conducted in Singapore (Liang et al., 2018) and Thailand (Krittayaphong and Permsuwan, 2018). With a 225-fold higher cost to that of enalapril, Sac-Val was not cost-effective compared to ACEIs in Thailand. Due to the relatively lower WTP (between SGD 20,000 to SGD 50,000) in Singapore, the price of Sac-Val has to be reduced by 32% to 70% for it to be cost-effective. Recently, a cost-effect evaluation of Sac-Val in South Korea (Park et al., 2019) concluded that Sac-Val is cost-effective compared to ACEIs, although the ICER was higher than that in our study.

The actual cost of a drug is a key factor in cost-effectiveness analysis. Since the fixed period for the negotiated price of Sac-Val in China is two years (01/2020-12/2021), a two-way sensitive analysis was further conducted by extending its cost range to $150 per-month, which simulated the conditions after the contract period. Given the current costs of enalapril at $0.9–$12.78 per-month, Sac-Val will have to cost $37.25–$48.44 or $113.91–$125.10 per-month to be considered cost-effective with WTP threshold of $10,276 or $30,828 respectively. Based on these results, we recommend that the price of ARNIs should be controlled after the negotiation period as well to increase its affordability. Furthermore, the price increment of Sac-Val should be kept less that sevenfold from the current cost of $17.12 per day, in order to be cost-effective over ACEIs in HF treatment.

According to the PARADIGM trial, Sac-Val is superior to enalapril in terms of reducing the risks of death and hospitalization of HF. In this analysis, the ICER displayed sensitivity to the risk and probability of CV death but was barely affected by the risk of hospitalization. This is consistent with some cost-effectiveness analyses of Sac-Val reported previously (King et al., 2016; Liang et al., 2018; Zueger et al., 2018). Given that HF is a chronic disease with significant mortality, especially in the elderly patients, the modeled population would decline significantly over the projected period due to increasing deaths. As a result, the benefit of Sac-Val in averting hospitalization might diminish. Furthermore, the lower costs of hospitalization and readmission in China compared to that in some developed countries might further offset the effect of the risk of hospitalization.

The majority of the patients included in the PARADIGM-HF were of NYHA class II with mild symptoms. Although the treatment-related inputs used in the model were obtained from the PARADIGM-HF population, we performed a base-case analysis to assess the cost-effectiveness in the high-risk population. When the proportion of NYHA class III and IV patients were respectively increased to 45% and 30% in the model, in order to simulate the epidemiological characteristics of HF in China, the ICERs increased over the 5, 10, and 20-year horizon. According to the results of PARADIGM-HF, the effect size for Sac-Val relative to enalapril varied depending on the baseline risk of CV death or HF-related hospitalization (Simpson et al., 2015). Due to the potentially greater benefit in higher-risk patients, Sac-Val was predicted to be more cost-effective among patients with a higher baseline risk (King et al., 2016). However, we observed a significant decrease in QALYs with a slight reduction in total costs, which eventually increased the ICERs in the high-risk population. This is consistent with a previous report and may attribute to the significant loss of quality of life in NYHA III and IV HF patients. Given the lower ICERs in the PARADIGM-HF population, we recommend the widespread use of Sac-Val in the relatively low risk population.

There are several limitations in our study that ought to be addressed. First, the treatment benefits of Sac-Val in this model were derived directly from the PARADIGM-HF trial. Although well designed clinical trials provide robust evidence, the benefits might still be overestimated compared to that in real-world practice. Second, the proportion of high-risk patients is higher in China compared to that in the PARADIGM-HF cohort, which may influence our results if real-world evidence is included. Third, although Asia-Pacific cohort was included in PARADIGM-HF, the raw data in Asian subgroup was absent, which can result in systematic bias and limit the validity of the results. Fourth, due to the lack of long-term follow-up data, the trends observed in PARADIGM-HF, such as improved CV mortality and reduced HF-related hospitalization, were extrapolated beyond the end of the trial period. This is a common limitation shared by most economic evaluation studies. Furthermore, the adverse events and related discontinuation of drug administration were not considered in this study. Sac-Val was associated with higher rates of hypotension and non-serious angioedema. Although adverse events reported in PARADIGM-HF trial were rarely required special treatment, the results from our analysis may be conservative.

## Conclusions

We reported the long-term cost-effectiveness of Sac-Val compared to enalapril for patients with HFrEF in China. Our findings offer new insights into the cost-effectiveness of novel ARNIs for HFrEF treatment and will enable informed decision making in China. Nevertheless, robust long-term and real-world estimates of cost-effectiveness are still needed.

## Data Availability Statement

The raw data supporting the conclusions of this article will be made available by the authors, without undue reservation, to any qualified researcher.

## Author Contributions

YW developed the study protocol, coordinated data collection, performed data analyses, reported study results and drafted the manuscript. ST collected data and revised the manuscript. PR, FZ, YC, and XG developed the study protocol. BZ supervised the work. All authors contributed to the article and approved the submitted version.

## Conflict of Interest

The authors declare that the research was conducted in the absence of any commercial or financial relationships that could be construed as a potential conflict of interest.
